# The transcriptome of *Balamuthia mandrillaris* trophozoites for structure-guided drug design

**DOI:** 10.1038/s41598-021-99903-8

**Published:** 2021-11-04

**Authors:** Isabelle Q. Phan, Christopher A. Rice, Justin Craig, Rooksana E. Noorai, Jacquelyn R. McDonald, Sandhya Subramanian, Logan Tillery, Lynn K. Barrett, Vijay Shankar, James C. Morris, Wesley C. Van Voorhis, Dennis E. Kyle, Peter J. Myler

**Affiliations:** 1 Seattle Structural Genomics Center for Infectious Disease (SSGCID), Seattle, WA USA; 2 Center for Global Infectious Disease Research, Seattle Children’s Research Institute, Seattle, WA USA; 3 Center for Tropical and Emerging Global Diseases, University of Georgia, Athens, GA USA; 4 Center for Emerging and Re-Emerging Infectious Diseases (CERID), Division of Allergy and Infectious Diseases, Department of Medicine, University of Washington, Seattle, WA USA; 5 Clemson University Genomics and Bioinformatics Facility, Clemson University, Clemson, SC USA; 6 Center for Human Genetics, Clemson University, Greenwood, SC USA; 7 Eukaryotic Pathogens Innovation Center, Department of Genetics and Biochemistry, Clemson University, Clemson, SC USA; 8 Department of Microbiology, University of Washington, Seattle, WA USA; 9 Department of Global Health, University of Washington, Seattle, WA USA; 10 Department of Pediatrics, University of Washington, Seattle, WA USA; 11 Present Address: Department of Pharmaceutical and Biomedical Sciences, College of Pharmacy, University of Georgia, Athens, GA USA

**Keywords:** Parasite genomics, Phenotypic screening

## Abstract

*Balamuthia mandrillaris*, a pathogenic free-living amoeba, causes cutaneous skin lesions as well as granulomatous amoebic encephalitis, a ‘brain-eating’ disease. As with the other known pathogenic free-living amoebas (*Naegleria fowleri* and *Acanthamoeba* species), drug discovery efforts to combat *Balamuthia* infections of the central nervous system are sparse; few targets have been validated or characterized at the molecular level, and little is known about the biochemical pathways necessary for parasite survival. Current treatments of encephalitis due to *B. mandrillaris* lack efficacy, leading to case fatality rates above 90%. Using our recently published methodology to discover potential drugs against pathogenic amoebas, we screened a collection of 85 compounds with known antiparasitic activity and identified 59 compounds that impacted the growth of *Balamuthia* trophozoites at concentrations below 220 µM. Since there is no fully annotated genome or proteome of *B. mandrillaris*, we sequenced and assembled its transcriptome from a high-throughput RNA-sequencing (RNA-Seq) experiment and located the coding sequences of the genes potentially targeted by the growth inhibitors from our compound screens. We determined the sequence of 17 of these target genes and obtained expression clones for 15 that we validated by direct sequencing. These will be used in the future in combination with the identified hits in structure guided drug discovery campaigns to develop new approaches for the treatment of *Balamuthia* infections.

## Introduction

*Balamuthia mandrillaris* is a ubiquitous soil-dwelling amoeba that is the causative agent of granulomatous amoebic encephalitis (GAE)^1–4^. Similar to the other two major pathogenic free-living amoebas, *Naegleria fowleri* and *Acanthamoeba castellanii*, *B. mandrillaris* infections, though uncommon, have > 90% case fatality rate^5^. In the United States, 109 *Balamuthia* cases in both immunocompetent and immunocompromised individuals have been reported with at least twice that many cases worldwide, but these rates of GAE are likely to be underestimated due to historically poor diagnosis^6^. Nonetheless, awareness of potential cases has been on the rise^7,8^. In addition to diagnostic awareness, increasing rates of amoebic infections in northern regions of the United States could also be early indicators that recent emergence of these diseases might be associated with global warming^6^. In contrast to *Naegleria* infections that present and progress extremely rapidly after exposure, *Balamuthia* incubation times might be as long as several weeks to months and disease progression more subacute or chronic, increasing the opportunity for therapeutic intervention^9^. However, treatment options remain very limited and not very efficacious, leading to poor outcomes even with the correct diagnosis.

The recent development of an inexpensive and easily prepared media, as well as increasing interest in *B. mandrillaris* as a public health concern, has facilitated the development of robust high-throughput drug screening methods. Where low throughput methods restricted screening to ~ 10–20 drugs at a time, the new high-throughput methods allow rapid screening of hundreds to thousands of drugs simultaneously and direct comparisons of activity. In this study, we identify FDA approved drugs that could potentially be repurposed for therapy alone or in combination against *B. mandrillaris*. These drugs can also be used as leads for further structure-guided drug discovery (SGDD) exploration and in vivo efficacy studies.

Structure based drug discovery (SBDD) was originally devised in the mid 1980’s^10^. Advances in protein structure determination, less expensive and faster computer processing, and better prediction software have reduced the timeline to solve target structures and design specific and selective drugs^10^. SBDD has been mentioned in the amoeba literature as an attractive method to design selective enzymatic inhibitors that specifically target the parasite over the human host^11^, but only a small number of laboratories have actually applied this methodology to pathogenic free-living amoebas. Sterol biosynthesis has been the most attractive target since parasites utilize ergosterol over cholesterol for making cell plasma membranes with distinct host biosynthetic differences that could be selectively targeted^12–14^. Glucose metabolism is essential for parasitic cell viability. Milanes et al., recently targeted glucokinase in *N. fowleri*, and described *Nf*Glck specific inhibitors with minimal activity against human glucokinase in recombinant enzymatic functional studies^15^. Other studies have looked at targeting histidine or shikimate essential amino acid biosynthetic pathways in *Acanthamoeba* species, which the hosts cannot synthesize de novo*,* as parasite specific targets for drug intervention^16,17^.

The development of new compounds against *B. mandrillaris* in particular has been hampered by the paucity of genomic information. Though draft genomes have been published, no structural and functional annotation is currently available^18,19^. This information is essential for the design of new drugs by SGDD/SBDD, as that methodology requires information about the molecular structure of the target protein. Once the protein coding sequences are annotated on the genome, rapid selection of multiple drug targets can be performed, for example by homology searches with known drug targets, thus paving the way for combinational therapy, a broadly established strategy to minimize the risk of drug resistance. This study presents the first proteome of *B. mandrillaris* reconstructed from RNA sequencing of logarithmic growing trophozoites, the infective form of the amoeba. Potential drug targets identified through phenotypic screening were selected specifically from the trophozoite transcriptome and PCR amplified. The clones were further validated by direct sequencing, providing the first step for recombinant expression and crystallization by the Seattle Structural Genomics Center for Infectious Disease (SSGCID) high-throughput gene-to-structure pipeline^20^.

## Results

### Phenotypic screens

We performed a drug susceptibility screen of 85 known anti-parasitic compounds against the trophozoite stage of *B. mandrillaris* and discovered that 59 of these compounds had 50% inhibitory concentration (IC_50_) efficacy at ≤ 220 µM concentration (Table 1). Our results indicated that only dequalinium chloride possessed nanomolar potency and that 43% of the currently recommended drugs by the CDC for treating *Balamuthia* infections appeared to be only moderately to slightly efficacious, with IC_50_ values ranging from 18.35 µM (pentamidine) to > 163.25 µM (fluconazole). Miltefosine, the newest drug addition to the amoebae chemotherapy cocktail, was inactive at the maximum screening concentration of 122.68 µM. Thus, all currently used therapeutics fall into the unacceptable activity range for today’s standards of hit to lead drug discovery and development. Macrolides (azithromycin, clarithromycin, roxithromycin, spiramycin A) have previously demonstrated potent activity against *Naegleria* or *Acanthamoeba* but did not show activity against *Balamuthia* at the concentrations we tested (IC_50_ values > 59 µM). Although we identified other ribosomal protein inhibitors for 50S (valnemulin, clindamycin, and solithromycin), 23S of the 50S [mirincamycin (cis- and trans-)], and the 16S ribosomal subunits (paromomycin) suggesting protein synthesis would be a useful target for drug therapy against *Balamuthia* infections. As pentamidine is currently used within the treatment regimen, we screened several anti-protozoal amidines, identifying hexamidine, octamidine and propamidine (IC_50_ values 4.46–6.5 µM) as being active against the amoeba. Although the exact mechanisms are still unknown, it is suggested that these compounds interfere with glyceraldehyde 3-phosphate dehydrogenase or interfere with nucleases. We further identified several 3-hydroxy-3-methylglutaryl-Coenzyme A reductase (HMGR) inhibitors (fluvastatin, atorvastatin, and simvastatin), with activity ranging from 1.18 to 3.03 µM.

**Table 1:** Phenotypic analysis of 85 compounds against logarithmic trophozoites in vitro (N = 2).

Compound	IC_50_ (µM) ± SEM	Compound	IC_50_ (µM) ± SEM
Dequalinium chloride	0.26 ± 0.05	Pyrimethamine	52.15 ± 2.01
Chlorhexidine	1.00 ± 0.09	Sitamaquine	52.45 ± 2.57
Fluvastatin sodium	1.18 ± 0.24	5-Fluorouracil	56.63 ± 2.72
Atorvastatin	1.26 ± 0.25	Promethazine	66.79 ± 8.93
HSP990	1.80 ± 0.02	Dyclonine HCL	81.27 ± 1.51
Simvastatin	3.03 ± 0.31	Sulconazole	81.51 ± 44.20
Hexamidine	4.46 ± 0.54	Dibucaine HCL	83 ± 0.65
WR 99210	4.93 ± 0.08	Terbinafine	83.25 ± 26.05
Octamidine	5.19 ± 0.01	Flucytosine ∆	86.34 ± 17.60
PHMB	5.84 ± 1.60	Desipramine	88.87 ± 1.67
Propamidine	6.50 ± 0.63	Sinefungin	91.01 ± 4.83
Valnemulin	11.79 ± 1.12	Allopurinol	92.17 ± 3.31
PS-15 (WR 250417)	14.02 ± 1.14	Floxuridine	93.18 ± 3.34
Benzalkonium chloride	14.09 ± 0.07	Primaquine	101.75 ± 7.94
Oligomycin B	14.92 ± 0.97	Tubercidin	123.17 ± 0.23
JPC 2056	15.13 ± 0.43	Fluridone	220.80 ± 2.87
Radicicol	15.22 ± 1.51	Caspofungin	> 45.73
Trans-mirincamycin	15.42 ± 2.84	Amphotericin B ∆	> 54.11
Mefloquine	16.54 ± 3.22	Spiramycin A	> 59.31
Domiphen bromide	17.08 ± 0.17	Roxithromycin	> 59.73
Auranofin	18.24 ± 0.57	Azithromycin	> 66.75
Pentamidine ∆	18.35 ± 1.47	Clarithromycin ∆	> 66.85
Cis-Mirincamycin	18.58 ± 1.70	Natamycin	> 75.11
Clindamycin	22.88 ± 1.05	Neomycin	> 81.35
Chlorpromazine	24.82 ± 1.78	Tafenoquine succinate	> 85.97
Solithromycin	29.66 ± 0.61	Lumefantrine	> 94.53
Ketoconazole	29.89 ± 10.62	Verapamil HCL	> 101.82
Pyronaridine tetraphosphate	33.56 ± 1.11	Fumagillin	> 109.04
Amodiaquine	33.58 ± 3.46	Sertaconazole	> 114.22
Asenapine	40.76 ± 6.16	Miltefosine ∆	> 122.68
Tioconazole	42.06 ± 28.45	Atovaquone	> 136.30
Difenoconazole	44.92 ± 19.00	Povidone–iodine	> 137.00
Halofuginone	46.08 ± 0.18	Voriconazole	> 143.14
Dihydroartemisinin	49.06 ± 2.72	Furosemide	> 151.17
Itraconazole	49.20 ± 21.66	Quinine	> 154.12
Posaconazole	49.57 ± 12.09	Chloroquine	> 156.31
Paromomycin	50.03 ± 0.50	Fluconazole ∆	> 163.25
Clotrimazole ∆	51.53 ± 23.21	Norflurazon	> 164.65
Climbazole	51.66 ± 13.23	Chlorpheniramine	> 181.96
Artesunate ∆	52.05 ± 4.42	Proguanil	> 197.06
		Glyphosate	> 295.73

Compounds annotated with ∆ have been previously used to treat GAE or cutaneous *Balamuthia* infections. The susceptibility is ranked in order of highly potent (left hand side column) to minimal potency (right hand side column) and the inhibitory concentration that causes 50% ATP depletion (death) is listed as the IC_50_ ± standard error mean (SEM). All compounds were initially screened from 50 μg/ml and converted to molarity for standardized testing.

### Transcriptome sequencing, assembly and functional annotation

#### Transcriptome assembly and proteome prediction

**Figure 1:**
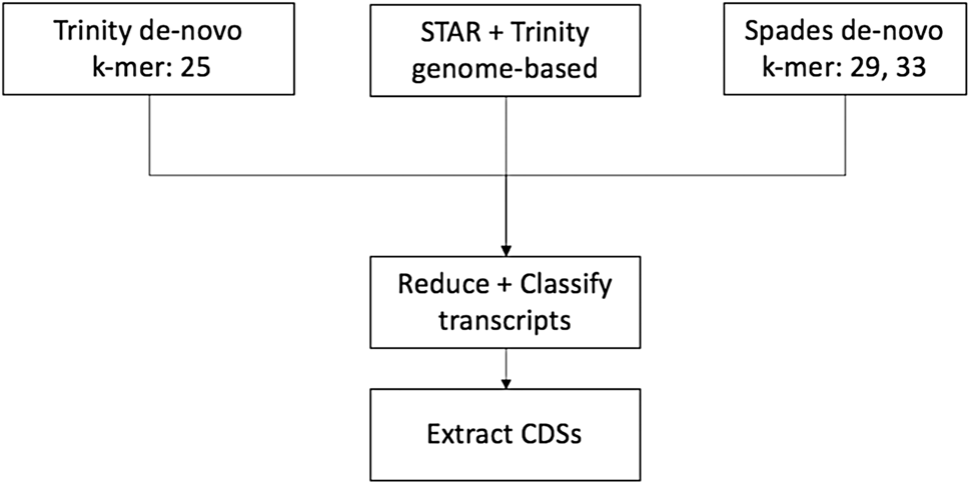
Overview of the main steps for predicting the *B. mandrillaris* proteome from RNA-seq reads using a hybrid approach. De-novo and genome-based assemblies are combined and processed with EviGene to reduce transcript redundancy and classify transcripts as encoding complete or incomplete protein coding sequences (CDSs, 5′ and/or 3′ truncated). CDSs are extracted, translated and annotated as “main” or alternate.

The sequencing of RNA isolated from an axenic laboratory culture of *B. mandrillaris* trophozoites yielded 30,473,902 paired-end reads (2 × 75 bp). We combined three de-novo assemblies, obtained from different assemblers and multiple k-mers, with a genome-based assembly based on the existing *B. mandrillaris* genome LFUI01^19^ and then predicted protein coding sequences (CDSs) with EvidentialGene (EviGene)^21^ (Fig. 1).

Of the 37 K transcripts with predicted CDSs, just over half (53%) translated to complete proteins. The top 1000 longest complete proteins averaged 1550 ± 423 amino acids in length, a number indicative of assembly quality that is roughly comparable to the AmoebaDBv44 *A. castellanii* proteome (1688 ± 539)^22,23^. The EviGene “main” sequences, representing the haploid proteome, contained 14 K proteins; though only 63% translated to complete proteins, they contain 90% of complete eukaryotic Benchmarking Universal Single-Copy Orthologs (BUSCOs), a standard measure to quantify the accuracy and completeness of assemblies (Table 2)^24^. Table 2 shows comparable levels of completeness for the transcriptome and the unannotated genome (LFUI01), but the EviGene “main” proteins stand out for containing only 2 duplicates among the complete BUSCOs.

**Table 2:** BUSCO quality and completeness assessment of the *B. mandrillaris* EviGene transcript assembly and predicted proteome compared to the draft genome (reference dataset for eukaryotes: N = 303).

	Assembled transcriptome		EviGene "main" proteins		Genome (LFUI01)	
# Input sequences	37,076		14,255		1605	
Complete BUSCOs	287	95%	270	89%	271	89%
Single-copy	*86*	*28%*	*268*	*88%*	*166*	*55%*
Duplicated	*201*	*66%*	*2*	*1%*	*105*	*35%*
Fragmented BUSCOs	4	1%	6	2%	10	3%
Missing BUSCOs	12	4%	27	9%	22	7%

Italic values describe Complete BUSCOs (single-copy + duplicated).

#### Proteome comparisons

**Figure 2:**
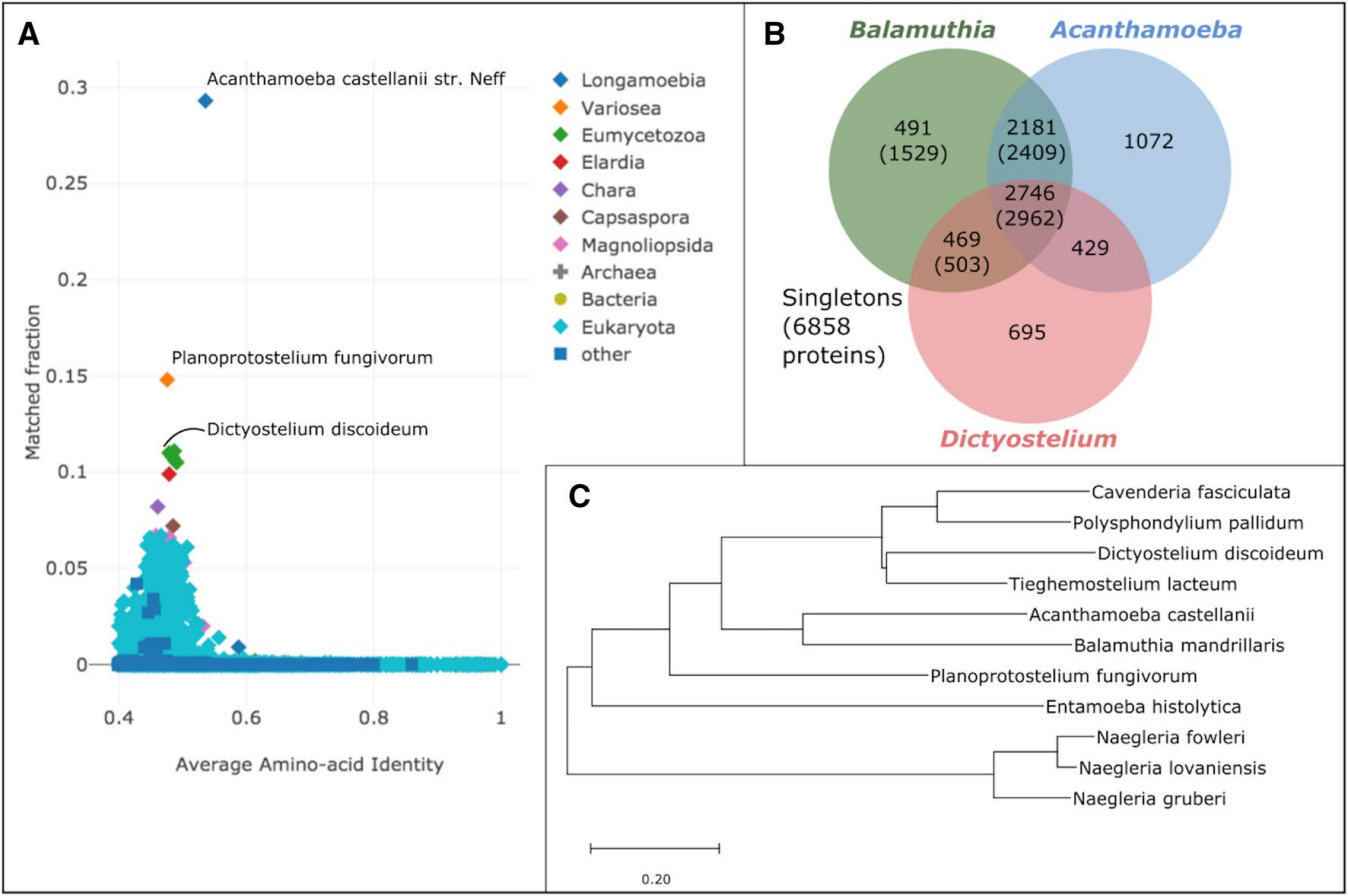
(**A**) AAI-profiler scatterplot. Shown are UniProt species with greater than 40% average amino-acid identity to the *Balamuthia* ‘main’ proteins. The species name of the top three proteomes with the largest fraction of matches to *Balamuthia* are indicated. (**B**) Venn diagram showing the overlap between orthologous cluster groups in the proteomes of *B. mandrillaris*, *A. castellanii* and *D. discoideum*. Total numbers of *B. mandrillaris* proteins in each group are in parenthesis. (**C**) Neighbor-joining tree illustrating the phylogenetic relationships between the proteomes of *B. mandrillaris* and closest species. The tree is based on alignment-free comparisons of the closest complete proteomes detected by AAI-profiler with three *Naegleria* species as outgroups.

We compared the EviGene ‘main’ proteins to other species in the UniProt database with AAI-profiler; it retrieved *A. castellanii* strain Neff as the closest sequenced proteome with 29% of matched fraction (Fig. 2A). Note that the AAI-profiler does partial sampling as it relies on SANSparallel, a fast homology search that is only as sensitive as BLAST above ca. 50% sequence identity^25^. We confirmed this result with a BLASTP search of the EviGene ‘main’ proteins against *A. castellanii*: it returned hits for 65% of the *Balamuthia* sequences, with an average identity of 44%, but matched 29% with at least 50% identity.

To gain further insight on how closely the two proteomes are related, we performed orthologous cluster analysis with *Dictyostelium discoideum* as the outgroup. Results show that 38% of the *Balamuthia* proteins cluster with *Acanthamoeba,* of which 21% are shared between the three species (Fig. 2B). This result and the high proportion of singletons (48%) highlights the divergence of *Balamuthia* from *Acanthamoeba*.

To place the *Balamuthia* proteome in an evolutionary context, we constructed a neighbor-joining tree from an alignment-free comparison of complete proteomes from selected Amoebas, with the non-Amoebozoa *Naegleria* as outgroup (Fig. 2C). As detected by AAI-profiler, the Discosea genera *Balamuthia* and *Acanthamoeba* are in a separate group from the Variosea genus *Planoprotostelium* and the Eumycetozoa Dictyostelids *Cavenderia*, *Polysphondylium*, *Tieghemostelium* and *Dictyostelium*. The Evosea genus *Entamoeba* is in a separate branch from the other Amoebozoa in the tree.

#### Functional annotation of the draft proteome

**Figure 3:**
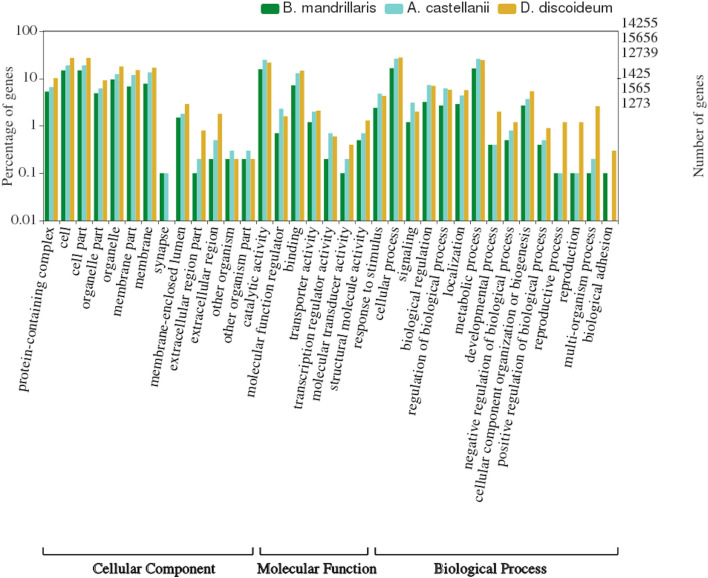
Level 2 top GO annotations. *B. mandrillaris* proteins (dark green) vs *A. castellanii* (cyan) and *D. discoideum* (yellow) as percentage of genes and total number of genes on a log(10) scale, significant relationships p-value < 0.05.

To characterize the proteome further and expand the pool of potential targets, we conducted preliminary functional annotations of the draft proteome dataset. Functional annotation of the EviGene “main” protein sequences with PANNZER2, one of the top-10 rapid methods in the CAFA2 NK-full benchmark, provided 26% of the sequences with a description and 25% with a lower level GO molecular function term. A plot of high-level GO terms compared with those obtained for *A. castellanii* and *D. discoideum*, one of the most thoroughly annotated amoebas in UniProt, shows a similar profile for the three species, with differences limited to smaller gene families representing less than 1% of the genes (Fig. 3). In terms of potential targets for SBDD, the *Balamuthia* GO annotations classified 284 (2%) of proteins as having kinase activity, of which over half (163) were classified as protein kinases, but further analysis is needed to target the kinome with accuracy^26^.

#### Target identification and validation

For this study, putative protein kinase targets from the phenotypic screens were left out, leaving a total of 25 potential targets (out of the original list of 52), to which we added 6 potential drug targets requested by the amoeba community via the gene-to-structure service portal of the SSGCID website. From this list of 31 protein names, 19 could be assigned to specific human protein sequences. A total of 14 *Balamuthia* sequences for 13 targets (there are 2 copies of topoisomerase II) were identified from a BLASTP search with the human sequences: 12 from the phenotypic screens, and 2 community targets. Average pairwise identity was 49% with 77% coverage. Another three of the community targets were not detected by BLASTP searches of the *Balamuthia* proteome using the human sequences, therefore *Acanthamoeba* sequences were used instead. This yielded a total of 17 *Balamuthia* sequences that were entered into the SSGCID gene-to-structure pipeline. Truncations around putative catalytic domains were designed for 9 of the 17 sequences to increase crystallization likelihood, leading to 23 constructs as cloning candidates. PCR amplification produced clones for 18 constructs. Direct sequencing was successful for 15 of these and sequence comparison with the “main” proteins from the EviGene assembly showed excellent matches with over 99% average amino acid identity, corresponding to 2 amino acid variations on average per sequence, and 100% coverage for all but the two largest proteins (84% coverage and 100% identity for the 1068 amino-acid long Exportin-1, 81% coverage and 99% identity for the 784-residue primase and C-term domains of topoisomerase II).

**Figure 4:**
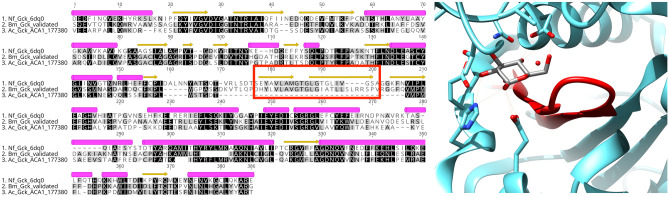
Sequence conservation of glucokinase in 3 pathogenic amoebas. Potential mis-assembly of the *A. castellanii* glucokinase (AmoebaDB ACA1_177380) highlighted on a multiple sequence alignment with the *B. mandrillaris* validated sequence (this study) and *N. fowleri* crystal structure (PDB: 6DA0)^15^; helical regions are annotated as pink tubes and beta-sheets as yellow arrows. The alignment was obtained with T-Coffee-Expresso^27^. The double-stranded beta-sheet missing in *A. castellanii* glucokinase is colored in red on the active site of the *B. mandrillaris* structure (PDB: 6VZZ).

The identity between the 15 validated protein sequences and their closest *A. castellanii* homologue ranged between 56 to 88%, with 3 notable exceptions: exportin-1 (21% identity), lanosterol 14-alpha demethylase (CYP51A) (28% identity) and glucokinase (51% identity). In the case of exportin-1, a multiple sequence alignment indicated that the *Balamuthia* protein was over 50% identical to the *N. fowleri* and *Planoprotostelium fungivorum* proteins, suggesting potential mis-assembly in the *A. castellanii* genome. Similarly, the *Acanthamoeba* glucokinase sequence appears to have a large deletion of over 30 residues compared to the *Balamuthia* and *Naegleria* sequences. This region corresponds to a double-stranded beta-sheet that lies in the glucose binding pocket in the *Naegleria* structure, and we would expect it to be conserved in *Acanthamoeba* (Fig. 4)^15^.

**Figure 5:**
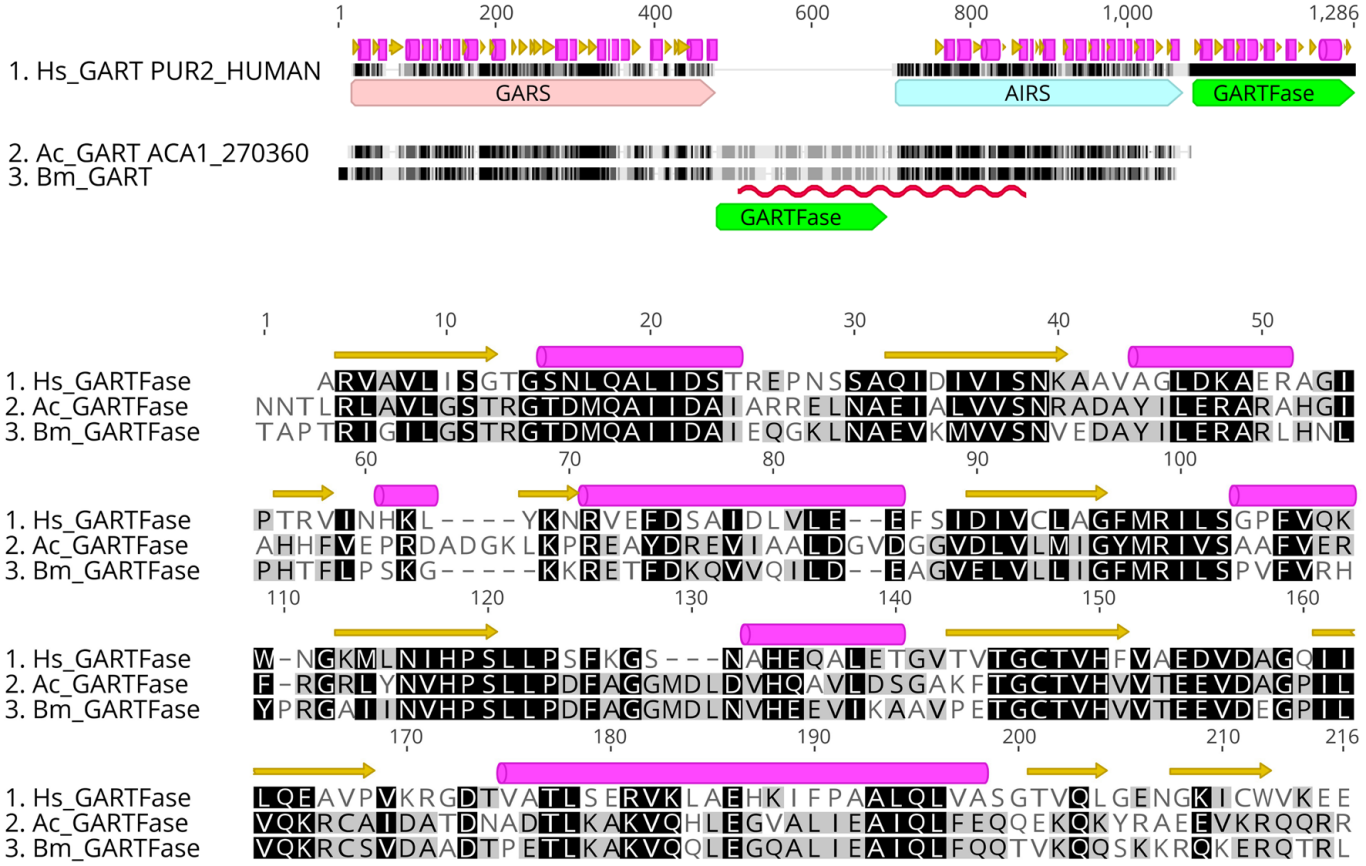
Top: Domain arrangement in human, *A. castellanii* and *B. mandrillaris* GART. Domains are annotated as large arrows on the alignment and higher level of residue conservation is represented as darker shades of gray. The region validated by direct sequencing in *Balamuthia* is underlined with a red squiggle. Secondary structure elements from human GART crystal structures are taken from UniProt. Bottom: Alignment of the GARTFase domains extracted from the GART sequences above.

Of the 13 targets that were also found in humans, five shared over 55% sequence identity overall to their human counterpart and might potentially have similar active sites: *S*-adenosyl-homocysteinase (SAHH), 3-hydroxy-3-methylglutaryl-coenzyme A reductase (HMGR), heat-shock protein 90-alpha (HSP90ɑ), histone deacetylase 1 (HDAC1) and exportin-1 (XPO1) (Table 3). As a consequence, SBDD for these targets will likely require exploration of potential alternate binding sites that are specific to the *Balamuthia* protein. We would expect selectivity to be more readily achievable for the other targets, with the topoisomerase ATPases as borderline cases. One promising example of a *Balamuthia* target that can be selectively targeted is the GARTFase domain of trifunctional purine biosynthetic protein adenosine-3 (GART). *Balamuthia* GARTFase has a low sequence identity to the human enzyme (37%) and has a different domain arrangement than in human GART. Whereas GARTFase is the C-terminal domain of human GART, it is the middle domain in *Balamuthia* (Fig. 5). This domain arrangement, confirmed by direct sequencing and conserved in *Acanthamoeba*, leads us to postulate that targeting double domains in GART may offer a promising avenue to develop drugs against those pathogenic amoebas.

**Table 3:** Sequence similarity (BLASTP) between *Balamuthia* validated sequences and UniProt identifiers of closest human counterpart. ^a^Lower than expected coverage due to incomplete sequencing of the clones. ^b^Additional targets selected by the community. Note that homology to human Glucokinase was too low to be detected with BLASTP at the chosen E-value (1e−3).

Balamuthia target	Pairwise identity	Target coverage	Closest human protein
*S*-adenosyl-l-homocysteine hydrolase^b^	58%	98%	sp|P23526|SAHH_HUMAN
Histone deacetylase 1	73%	83%	sp|Q13547|HDAC1_HUMAN
Lanosterol 14-alpha demethylase (CYP51A)	26%	96%	sp|Q16850|CP51A_HUMAN
Methionyl-tRNA synthetase (methionine tRNA ligase) (MetRS)^b^	54%	79%	sp|P56192|SYMC_HUMAN
Heat shock protein HSP90-alpha	69%	100%	sp|P07900|HS90A_HUMAN
Calcium ATPase, haloacid dehydrogenase (HAD) domain	43%	100%	tr|A0A0A0MSP0|ATP2C2_HUMAN
3-Hydroxy-3-methylglutaryl-CoA reductase (HMG-CoA reductase) (HMGR)	62%	97%	sp|P04035|HMDH_HUMAN
Glucokinase^b^	–	–	None
DNA topoisomerase II copy 1, ATPase and transducer domains	55%	97%	sp|Q02880|TOP2B_HUMAN
DNA topoisomerase II copy 1, toprim and C-term domains	49%	99%	sp|P11388|TOP2A_HUMAN
DNA topoisomerase II copy 2, ATPase and transducer domains	52%	97%	sp|P11388|TOP2A_HUMAN
DNA topoisomerase II copy 2, toprim and C-term domains	39%	80%^a^	sp|Q02880|TOP2B_HUMAN
Exportin-1 (CRM1, XPO1)	57%	83%^a^	sp|O14980|XPO1_HUMAN
Xylose isomerase (xylA)^b^	–	–	none
Trifunctional purine biosynthetic protein adenosine-3 (GART), GARTFase domain	37%	82%	sp|P22102|PUR2_HUMAN

## Discussion

Based on previously determined in vitro activity and the few surviving cases of *Balamuthia* GAE infections, the Centers for Disease Control and Prevention (CDC) recommends that the drug cocktail regimen for treating disease include a combination of pentamidine, sulfadiazine, flucytosine, fluconazole, azithromycin or clarithromycin, and miltefosine^6^.

We thus proceeded to test these compounds first in order to confirm inhibition and assess if they have sufficient potency (< 10 µM) for hit-to-lead phenotypic drug screening. According to our screens, none of the recommended compounds passed this criterion. We therefore expanded our drug screening to other compounds that previously displayed anti-*Acanthamoeba*, anti-*Naegleria,* or anti-malarial activity, starting with the macrolides.

Our screening results consistently indicate that the compounds belonging to the macrolide drug class (azithromycin, clarithromycin, roxithromycin, and spiramycin) are inactive, in agreement with previous results^28^. Interestingly, solithromycin, a known ketolide antibiotic against macrolide-resistant Streptococcal species^29^, appeared to show moderate activity against *B. mandrillaris* (29.66 µm). We found that polyene antimycotics such as amphotericin B and natamycin, that target ergosterol within fungal cell membranes^30^, also were also inactive against *B. mandrillaris*. We then tested the azole compounds. CDC studies reported that fluconazole was inactive at concentrations lower than 10 µg/mL^31^. We were able to confirm the fluconazole result; however, other antifungal azoles (ketoconazole, tioconazole, difenoconazole, itraconazole, posaconazole, clotrimazole, climbazole, and sulconazole) displayed better, though still moderate, activity (29.89–81.51 µm). We tested flucytosine and miltefosine and both displayed moderate to poor activity against *B. mandrillaris* with an IC_50_ of 86.34 and > 122 µm, respectively. Flucytosine was previously described to inhibit 61% *Balamuthia* cytopathogenicity at 10 µg/mL (77 µm). Combined with our own results, this suggests flucytosine and miltefosine are equipotent^32^. Although miltefosine has been reported to have moderate activity at concentrations of 63–100 µm^32,33^, another study^34^ agrees with our data that miltefosine is less potent with a higher IC_50_ value > 122 µm. As for pentamidine, our results are consistent with prior studies that obtained comparable IC_50_ values between 9 and 29 µm^31,35,36^.

Thus, of the combination drug cocktail recommended by the CDC, only pentamidine and flucytosine appear to have in vitro activity against *B. mandrillaris*. It is possible that the recommended drug therapy is active in combination, and not when tested in isolation as in this study. It is also possible that the drugs are biologically activated in vivo or are only active against an in vivo form that we have not assayed. We therefore cannot rule out activity for the recommended drugs based solely on our in vitro sensitivity screens. Known inhibitors of 50S and 16S ribosomal proteins, such as macrolides and lincosamides, showed no or very poor activity in our screens, but we can only speculate that translation in *Balamuthia* evolved independently of prokaryotic endosymbiosis. Though *Parachlamydia acanthamoebae* was shown to infect *B. mandrillaris*^37^, we could find no evidence of *P*. *acanthamoebae* 50S ribosomal proteins in our *de-novo* transcriptome assembly.

We previously identified statins, which target 3-hydroxy-3-methylglutaryl-coenzyme A reductase (HMGR), as active compounds against *B. mandrillaris*^36^. As part of this study, we tested three additional statins: fluvastatin, atorvastatin and simvastatin and observed that they were active against *B. mandrillaris* at 1.18–3.03 µM concentration. Simvastatin and fluvastatin in particular have been shown to have better brain penetration compared to other statins^38^, indicating potential for drug repurposing. These results not only validate this drug class as promising lead molecules for the potential treatment of *B. mandrillaris* GAE, but also HMGR as a priority target of interest for future studies.

From a drug-repurposing standpoint, the compounds described in this study yielded a plethora of potentially useful drugs that act on *B. mandrillaris*. Available chemical inference data associated only few of the compounds with a specific protein target. Given our low success rate of structure determination in parasites, we increased the pool of potential protein targets by including results from our previous drug discovery studies. These include screening the MMV Malaria and Pathogen boxes that identified 11 compounds with equipotency to nitroxolone (8-Hydroxy-5-nitroquinoline) IC_50_ of 2.84 µM^32^, indicating these molecules could be used as an initial starting point for medicinal chemistry structure activity relationship (SAR) studies. More recently, we identified 63 compounds through screening the Calibr ReFRAME drug repurposing library, with activities ranging from 40 nM to 4.8 µM^36^.

Chemical inference from our previous drug screening efforts and the results from this study identified a total of 52 potential protein targets in *B. mandrillaris*. After excluding kinases, our target list was reduced to 25 proteins. Of these, 15 were identified solely from this study and the remainder was identified in several of our screening efforts. These 25 protein targets and an additional 6 community targets of interest were submitted for structural determination to the Seattle Structural Genomics Center for Infectious Diseases (SSGCID) gene-to-structure pipeline.

The first step of the SSGCID pipeline involves cloning of the *B. mandrillaris* sequences encoding the protein targets identified in the screens. However, lack of annotation of the *Balamuthia* genome hampered these efforts: although we were able to locate some sequences using BLAST searches of the *Balamuthia* genome using the human and *Acanthamoeba* homologues, PCR amplification from *B. mandrillaris* cDNA (or gDNA) was not successful. Therefore, transcriptome sequencing of *Balamuthia* was performed.

We reconstructed a draft *B. mandrillaris* proteome from a transcriptome that we obtained by combining 4 different assemblies from a single RNA-seq experiment. According to the BUSCO standard benchmark, our set of Evigene ‘main’ proteins is as complete (90%) as the reference genome, but with only a fraction of duplicates. We have thus based our subsequent analysis on this set as a representative of the haploid proteome of *B. mandrillaris* trophozoites. From our search of UniProt complete proteomes, *A. castellanii* stood out as the closest species with no other close relative, making the *Acanthamoeba* and *Balamuthia* proteomes the sole representatives of the phylum Discosea. As a third of the *B. mandrillaris* proteins appeared to be truncated, our phylogenetic analysis is based on an alignment-free method suitable for computing evolutionary distances between complete or incomplete proteomes^39^. The evolutionary tree placed the Discosea in a separate group as expected, highlighting their degree of divergence to the other pathogenic amoeba *N. fowleri*. Although they belong to the same phylum, *Acanthamoeba* and *Balamuthia* appear to be distantly related according to our alignment-based analyses-less than half of the proteins clustered together, and just above a quarter shared over 50% sequence identity.

We obtained functional annotation for above a quarter of the proteins using a conservative method. Annotation with the less stringent blast2go still leaves 60% of sequences with no annotation, a 20% higher proportion than the *Acanthamoeba* proteome. Much work remains to be done to characterize the complete proteome. Nonetheless, validation through direct sequencing showed that for our chosen targets at least, our assembly is accurate. In addition, alignment of the validated sequences to the genome revealed frameshifts in the reference assembly, which was further supported by analyzing the genome annotation we obtained with GENSAS^40^. Our attempts to correct the genome by re-assembling the publicly available PACBIO reads with modern assemblers and correcting exons with the transcripts were unsuccessful. Future efforts might succeed once the 454 Illumina reads used in the published genomic assembly will be made accessible.

The majority of the protein targets that we identified in *Balamuthia* had closely related sequence homologues in *Acanthamoeba*, a likely indication that they perform essential function in both organisms. From the high level of sequence identity, we would expect similar pattern of inhibition with the drugs tested in this report. However, our preliminary studies indicate that this is not the case. For example, the heat shock protein HSP90 is highly conserved in *Balamuthia* and *Acanthamoeba* (78% pairwise sequence identity), and yet we measured up to 5.56-fold differences in IC_50_ values in response to the same drug. Another example is 3-hydroxy-3-methylglutaryl-CoA reductase; the sequence identity is still high at 70%, but when we compared responses to nine different statins, we observed large differences ranging from > 2.51 to > 112-fold differences. The final example is lanosterol 14-alpha demethylase which displayed the least pairwise identity to *A. castellanii* protein sequence with 28.5% similarity. *B. mandrillaris* was found to be less susceptible to the 11 different azoles tested with 0.79 to 646-fold difference compared to the response of *A. castellanii*. The implication for drug discovery is that we cannot assume that orthologous sequences in *Balamuthia* and *Acanthamoeba* will be close enough to permit simultaneous targeting of these two pathogenic amoebas.

## Conclusion

Through drug susceptibility screening with known antiparasitic compounds against *B. mandrillaris*, we identified protein targets with potential for treating *Balamuthia* granulomatous amoebic encephalitis. The reconstruction and annotation of the draft proteome from RNA-seq allowed us to amplify, clone and validate the *B. mandrillaris* targets by direct sequencing.

The haploid proteome appears distantly related to that of its closest relative *A. castellanii*, and thus provides an essential resource for further drug discovery and biological investigation. Even in cases where the target sequences are conserved, our preliminary results indicate that potent inhibitors against *Acanthamoeba* or *Naegleria* failed to inhibit *Balamuthia* growth, suggesting that the quest for a broad-spectrum drug against all three pathogenic amoebas might prove elusive. Drugs will need to be specifically developed for each amoeba.

This study illustrates how the combination of phenotypic drug screening and a single RNA-seq experiment with short reads are enabling structure-based drug design against a eukaryotic pathogen with no prior proteome information. As there are currently no genetic tools available for *B. mandrillaris*, the results presented here will enable future studies to validate specific drug targets. Validation strategies may include co-crystallization of protein and inhibitors; development of drug resistant *B. mandrillaris* clones followed by whole genome sequencing; specifically targeting genes through RNAi (which has been used successfully in other free-living amoebae) or, in the era of CRISPR/Cas9-mediated gene editing, the generation of conditional knock outs, would be some promising ways of target validation for future studies within this pathogen.

## Materials and methods

### Cell culture

#### Maintenance of *Balamuthia mandrillaris*

The pathogenic *B*. *mandrillari*s (CDC:V039; ATCC 50209), a GAE isolate, isolated from a pregnant Baboon at the San Diego Zoo in 1986 was donated by Luis Fernando Lares-Jiménez ITSON University, Mexico^35^. Trophozoites were routinely grown axenically in BMI media at 37 °C, 5% CO_2_ in vented 75 cm^2^ tissue culture flasks (Olympus), until the cells were 80–90% confluent. For sub-culturing, 0.25% Trypsin–EDTA (Gibco) cell detachment reagent was used to detach the cells from the culture flasks. The cells were collected by centrifugation at 4000 rpm at 4 °C. Complete BMI media is produced by the addition of 10% fetal bovine serum and 125 μg of penicillin/streptomycin antibiotics. All experiments were performed using logarithmic phase trophozoites.

### Target identification

#### Phenotypic screening

We previously developed and standardized robust high-throughput screening methods for the discovery of active compounds against *B. mandrillaris* trophozoites^35^. The trophocidal activity of compounds were assessed using the CellTiter-Glo 2.0 luminescent cell viability assay (Promega, Madison, WI). In brief, *B. mandrillaris* trophozoites cultured in BMI-complete media were seeded at 16,000 cells/well into 96-well plates (Thermo Fisher 136102) with various compounds diluted in twofold serial dilutions to determine the 50% inhibitory concentration (IC_50_). The highest percentage of DMSO diluted in the highest screening drug concentration was 1%. Control wells were supplemented with 1% DMSO or 12.5 μM of chlorhexidine, as negative and positive controls, respectively. All assays were incubated at 37 °C for 72 h. At the end time point, 25 μL of CellTiter-Glo reagent was added to all wells. The plates were shaken using an orbital shaker at 300 rpm at room temperature for 2 min to induce cell lysis. After shaking, the plates were equilibrated at room temperature for 10 min to stabilize the luminescent signal. The ATP luminescent signal (relative light units; RLUs) were measured at 490 nm by using a SpectraMax i3X (Molecular Devices, Sunnyvale, CA). Drug inhibitory concentration (IC_50_) curves were generated using total ATP RLUs where controls were calculated as the average of replicates using the Levenberg–Marquardt algorithm, using DMSO as the normalization control, as defined in CDD Vault (Burlingame, CA, USA). Values reported are from a minimum of two biological replicates with standard error of the mean.

#### Selection of target genes

The protein names for verified potential targets were retrieved through Calibr at Scripps Research (https://reframedb.org/). The corresponding human protein sequences were downloaded from UniProt and queried against the *B. mandrillaris* assemblies using BLAST sequence similarity searches. Candidate targets were confirmed by comparing their protein sequences with closest sequence homologues in *Acanthamoeba* and *Naegleria* species and checking the *B. mandrillaris* functional annotation, where available. ORFs were selected for cloning from the Trinity de-novo assembly. Manual correction of putative start sites from multiple sequence alignments was performed with Geneious Prime 2019.1.1 (https://www.geneious.com).

### RNA extraction, library preparation and sequencing

#### RNA extraction

*Balamuthia mandrillaris* were cultured and harvested as described above; the cells were counted and adjusted to 2 million cells for each extraction. Total RNA isolated using the RNA extraction kit (Agilent) as per manufacturing instructions. In brief, to the pellet of *Balamuthia* cells, 350 μL of lysis buffer and 2.5 μL of β-mercaptoethanol were added and homogenized. This was transferred into a prefilter spin cup and centrifuged at maximum speed, 14,000×*g*, for 5 min. The filtrate was retained and an equal volume of 70% ethanol was added to the filtrate and vortexed until the filtrate and ethanol were mixed thoroughly. This mixture was then transferred into an RNA binding spin cup and receptacle tube and centrifuged at maximum speed for 1 min. The filtrate was discarded and 600 μL of 1 × low salt buffer was added and centrifuged at maximum speed for 1 min. The filtrate was removed and centrifuged at maximum speed for 2 min. DNase solution was added and incubated for 15 min at 37 °C. After incubation 600 μL of 1 × high salt buffer (contains guanidine thiocyanate) was added and centrifuged at maximum speed for 1 min. The filtrate was discarded and 300 μL of 1 × low salt buffer was added and centrifuged at maximum speed for 2 min. 100 μL of elution buffer was added and incubated at room temperature for 2 min. Final elution was into a sterile 1.5 mL microcentrifuge tube at maximum speed for 1 min.

Extracted RNA was stored at − 80 °C until further required. The integrity and purity of the RNA was assessed via RT-PCR and gel electrophoresis on a 2% agarose gel. The concentration was determined by measuring 280 nM absorbance on a nanodrop (Nanodrop 1000, Thermo Scientific).

RNA quality was reassessed after a freeze thaw cycle using the Bioanalyzer RNA 6000 pico chip (Agilent, 5067-1513) and quantity was assessed using the Qubit RNA Broad Range Assay (Invitrogen, Q10210). The mRNA was isolated using the NEB Poly(A) mRNA Magnetic Isolation Module (NEB, E7490S) and prepared using a version of the Stranded RNA-seq protocol that was modified for *Leishmania*^41,42^. Only the negative stranded RNA-seq library preparation portion was performed. Library quantity and quality was assessed using the Qubit dsDNA High Sensitivity Assay (Invitrogen, Q32851), Bioanalyzer High Sensitivity DNA Chip (Agilent, 5067-4627) and the KAPA library quantification kit (Roche, KK4824). Libraries were sequenced on the Illumina Hiseq 4000, yielding 2 × 75 bp paired end reads.

### Transcripts assembly and annotation

Reads were quality filtered with Trimmomatic and assembled *de-novo* with Trinity v2.8 (k-mer = 25) and Spades v3.13 (k-mer = 29 and 33) after clipping of the adaptor sequences^43–45^. Further, quality-filtered reads were aligned to the published *B. mandrillaris* genome LFUI01 with STAR v2.6 and assembled with Trinity^46^. The three assemblies thus obtained were combined with EvidentialGene v19jan01 (EviGene) with BUSCO homology scores as input for the classifier^21^. Throughout the analysis, BUSCO v3 analysis was performed on either the European or Australian Galaxy mirrors^47,48^. Assemblies were screened for vector and common eukaryotic contaminants with the EvidentialGene utilities asmrna_vecscreen and asmrna_trimvec. Functional descriptions and gene ontology (GO) annotations of the EviGene ‘main’ proteins were predicted with PANNZER2^49^. GO annotations that were highest ranked by PANNZER2 were visualized with WEGO 2.0^50^.

### Comparison to other species and phylogenetic analysis

Proteome comparisons to other species in the UniProt database were obtained from the AAI-profiler server^51^. Cluster analysis and Venn diagrams of orthologous clusters were generated with OrthoVenn2^52^ (e-value: 1e−5, inflation value: 1.5). Unless otherwise specified, all BLAST searches were performed with BLAST + v2.8.1 and an expectation value of 0.001^53^. Pairwise distances for alignment-free phylogeny reconstruction were calculated with Prot-SpaM^39^. Input sequences included the *Balamuthia* EviGene ‘main’ proteins (this study), AmoebaDB *A. castellanii* strain Neff, *N. fowleri* ATCC 30894 and *N. lovaniensis* Braker1 predicted proteins^54,55^, and UniProt complete reference proteomes (*C. fasciculata* UP000007797, *N. gruberi* UP000006671, *P. pallidum* UP000001396, *D. discoideum* UP000002195, *P. fungivorum* UP000241769 and *T. lacteum* UP000076078). Phylogenetic relationships were inferred by constructing a neighbor-joining tree from the word match-based Prot-SpaM distance matrix using MEGA X^56,57^.

### PCR and sequence validation

#### Cloning

All *B. mandrillaris* constructs were cloned, expressed, and purified using SSGCID established protocols^58,59^. The genes selected were PCR-amplified using cDNA template and purchased primers (Integrated DNA Technologies, Inc., Coralville, IA) (Supplementary Table S1). The amplicons were extracted, purified and cloned into a ligation-independent cloning pET-14b derived, N-terminal His tag expression vector pBG1861 with a T7 promoter^60^. The cloned inserts were then transformed into purchased GC-5 cells (Genesee Scientific, El Cajon, CA) for ORF incorporation. Plasmid DNA was purified from the subsequent colonies and further transformed in chemically competent *E. coli* BL-21(DE3)R3 Rosetta cells with a chloramphenicol restriction.

#### Sequence validation

Each *B. mandrillaris* construct was sequenced from both 5′- and 3′-ends with a custom forward primer (5′-GCGTCCGGCGTAGAGGATC-3′, 40nt upstream from the T7 promoter customary forward primer) and the T7 terminator reverse primer (5′-GCTAGTTATTGCTCAGCGG-3′) at GeneWiz (South Plainfield, NJ). The reads were assembled and matched to the expected sequences with the phrap assembler and cross_match^61^. Translations of the longest ORF in all six frames of the consensus sequence (or the forward read if unassembled) were then aligned using MUSCLE^62^ with the SSGCID target protein sequence to determine the best translated protein sequence and its alignment, percent identity and percent coverage. Manual examination of the sequences and alignments was performed in Geneious.

## Supplementary Information

Supplementary Information.

## Data Availability

Illumina raw reads have been deposited at the National Center for Biotechnology Information (NCBI) BioProject repository with the accession number SRR12006108 under project PRJNA638697. This Transcriptome Shotgun Assembly project has been deposited at DDBJ/EMBL/GenBank under the accession GISS00000000. The version described in this paper is the first version, GISS01000000. The annotated protein sequences from the EviGene ‘main’ assembly are available on NIH Figshare under https://doi.org/10.35092/yhjc.12478733.v1. All data that are associated with the drug susceptibility study are archived using the database from Collaborative Drug Discovery (CDD; http://www.collaborativedrug.com/). The CDD database accommodates both compound chemistry data and results from phenotypic or target activity, cytotoxicity screening, and computed properties. The CDD database is becoming an established standard for the sharing of data within this community, and we are eager to facilitate the distribution of our results in a similar manner.
